# A comparative analysis of traditional classroom and future classroom lab in mathematics education: exploring pre-service teachers’ emotions and self-efficacy beliefs

**DOI:** 10.3389/fpsyg.2026.1870540

**Published:** 2026-09-14

**Authors:** Ana Isabel Montero-Izquierdo, Jin Su Jeong, David González-Gómez

**Affiliations:** Department of Science and Mathematics Education, Teacher Training College, University of Extremadura, Cáceres, Spain

**Keywords:** mathematics education, future classroom lab (FCL), pre-service teachers (PSTs), emotions, self-efficacy beliefs, traditional classroom (TC)

## Abstract

This study addresses the lack of empirical research regarding how Traditional Classroom (TC) versus innovative Future Classroom Lab (FCL) learning environment influence students’ emotional responses and self-efficacy beliefs in mathematics education. A quasi-experimental design using intact classroom groups was employed to accommodate scheduling constraints and ensure equitable access to the newly pedagogical approaches. Therefore, the sample was selected by the occasional sampling method by convenience. A total of 105 Pre-Service Teachers (PSTs) participated in the study. The instrument was administered online before and after the intervention. The questionnaire comprised 23 items: 14 assessing achievement emotions and 9 assessing self-efficacy beliefs in mathematics learning. The instruments demonstrated robust internal consistency, Cronbach’s alpha coefficients exceeded 0.70. The ANOVA indicated significant effects on emotions and self-efficacy beliefs, with larger effects when the course intervention was implemented in the FCL setting. When gender and educational background were assessed as covariates, no significant effects of these variables were observed. Post-intervention correlations between emotions and self-efficacy beliefs were higher in the FCL (76.2%) than in the TC (23.8%). The main objective of this study was to examine how transitioning from a TC setting to a FCL impacts PSTs’ emotions and self-efficacy beliefs. Finally, the results point at that the integration of FCL in this mathematics proposal significantly reduces the emotional aversion and low self-efficacy in mathematics that PSTs typically exhibit toward this subject. Therefore, this study can positively potentially impact PSTs’ learning and their future teaching practices as a direct consequence of utilizing the FCL in mathematics education.

## 1 Introduction

Pre-service teachers frequently exhibit emotional rejection and low self-efficacy toward mathematics. This study addresses a critical research gap by incorporating innovative learning environments, such as the Traditional Classroom (TC) versus the Future Classroom Lab (FCL), thereby allowing for an empirical evaluation of whether these settings improve their predisposition. Demonstrating a positive effect is essential to ensure that future teachers gain the confidence and affective security required for successful practice in mathematics education.

Over the past decade, mathematics educators have been exploring alternative teaching methods that are more effective than traditional approaches (Frejd and Muhrman, 2020). Many external factors support or hinder this transition, including the physical learning spaces, which have recently attracted international interest (Bennett, 2021). Transitioning from traditional teaching methods to innovative approaches also requires a transformation of the classroom, making them more adaptable and appealing, thereby fostering student-centered learning (Yue, 2024). The classroom space as well as its organization and available resources influence teachers’ selection and application of pedagogical methodologies (Martin, 2002). A traditional mathematics classroom is often organized in a teacher-centered approach, with desks arranged in rows facing the blackboard. In contrast, in innovative learning spaces such as the FCL, which is organized in learning spaces, a learner-centered approach can be applied. In this classroom setting, students can move and interact and appear to affect their motivation to learn, which may indirectly impact learning outcomes (Stadler-Altmann, 2015). Furthermore, the arrangement of the physical learning space is an important factor to consider optimizing learning. It was found that a more dynamic and flexible space positively impacted student learning (Imms and Byers, 2017; Peng et al., 2022).

In the previous decade, a variety of innovative learning environments have been introduced across diverse educational disciplines globally. Innovative learning space facilitates pedagogical adjustments and promotes learning experiences that increase students’ interest, engagement (Ryan and Patrick, 2001), and academic performance (Tanner, 2009). Specifically, Byers et al. (2018) found that students in an innovative learning space performed better academically in mathematics than their peers with similar cognitive abilities in a traditional classroom. Furthermore, these types of learning spaces are correlated with improved student engagement and motivation in learning (Ryan and Patrick, 2001), and with students’ mathematical performance (Byers et al., 2014; Imms and Byers, 2017). These shifts in classroom settings pose new questions that require teacher training in active pedagogies, digital competence and in innovative settings (Lau and Jong, 2023; Lessard et al., 2015). One type of innovative learning space that has appeared in recent years is called the FCL, which was created in 2012 by European Schoolnet (Dúo-Terrón, 2024).

Moreover, learning spaces may trigger diverse emotions among students. Consequently, it is imperative to investigate students’ emotions within academic settings. Emotion plays a crucial role in the learning process, and there is a discernible correlation between students’ emotional states and their perception of the classroom environment (Frenzel et al., 2007; Li et al., 2020). Pekrun (2006) identified that students can experience positive emotions when they engage in autonomous work and problem-solving tasks (Pekrun, 2006). In addition, cooperative learning and positive emotions are positively correlated, while students who do not engage with one another have more negative feelings (Jeong and González-Gómez, 2026; Nummenmaa and Nummenmaa, 2008). Most research in the field of cognitive psychologists has focused on how students learn; they did not consider the environment or how students relate to one another (Chiang and Liu, 2014). Additionally, research has demonstrated a positive connection between emotion and memory, and instructional practices that aim to evoke good emotions in learners have been found to be successful (Hertel and Parks, 2002). Moreover, emotions are important when learning mathematics because they have a substantial effect on students’ motivation and academic achievement (Schoenherr et al., 2025).

Another important affective variable that influences learning is self-efficacy belief (Hoy et al., 2009). Some research suggests that positive self-efficacy beliefs in mathematics have been related to the classroom environment (Gilbert et al., 2014). Students’ self-efficacy can contribute to the enhancement of positive emotions and the reduction of negative ones (Caprara et al., 2022). According to Løvoll et al. (2017), positive emotions not only directly encourage academic engagement but also sustain it by boosting intrinsic motivation. In general, self-efficacy beliefs are influenced by affective domains: while positive moods increase self-efficacy beliefs and promote positive thoughts, negative moods elicit negative thoughts and decrease self-efficacy beliefs (Medrano et al., 2016; Schutte, 2014). According to literature, learners who believe in their own abilities can manage better stressors, use their personal resources more effectively, have more optimistic expectations, and set higher goals (Jeong and González-Gómez, 2025; Luszczynska et al., 2005; Schwarzer and Hallum, 2008).

### 1.1 Objectives of the study and research questions

The importance of analyzing emotional factors and self-efficacy in Pre-Service Teachers’ (PSTs) development lies in the fact that these psychological constructs directly shape their future instructional practices and, consequently, their students’ academic achievement in mathematics. Consequently, the primary objective of this study is to examine how transitioning from a TC setting to a FCL impacts pre-service mathematics teachers’ emotions and self-efficacy beliefs. To achieve this objective, this study addresses the following research questions:

RQ1: Does the learning setting influence pre-service teachers’ emotions when applying for a mathematics education intervention?

RQ2: Does the learning setting influence pre-service teachers’ self-efficacy beliefs when applying for a mathematics education intervention?

RQ3: Is there a difference in correlations of the pre-service teachers’ emotions and self-efficacy beliefs depending on the learning setting where the mathematical intervention was applied?

## 2 Literature review

### 2.1 Traditional and innovative learning environments

Recent studies on educational innovation have tended to concentrate on how instructional strategies, technological aids, and classroom environments can enhance student learning and academic performance (Nurmi and Kiuru, 2015; Lewohl, 2023). Over years, lectures in a TC setting are characterized by the hierarchical order of the classroom, which provides a spatial foundation for teachers to unilaterally transmit knowledge to students and the memorization of content (Ashworth et al., 2004). The pedagogical design of the TC has a fixed configuration; the teacher is placed in the front and presents the content of the subject. The rigidity of TC arrangement prevents teachers from applying methodologies that facilitate student-centered learning enhanced by technology, as advocated in current educational global policy (Amiri, 2025; Dovey and Fisher, 2014; Yadav, 2026).

According to some authors, TCs tend to favor pedagogies associated with superficial learning experiences rather than the processes necessary for deep learning (Dumont and Istance, 2010; Hattie and Donoghue, 2016), and the inflexible design limits the ability of teachers to apply methodologies more likely to support the student-centered learning and use of technologies (Dovey and Fisher, 2014; Imms and Kvan, 2021). The transformation of educational environments along with the use of technology is a current concern for many institutions (Atif, 2013) and diverse educational paradigms have emerged in recent decades to change and meet the demands of current society (Espíndola Juárez and Granillo Macías, 2021; Yadav, 2026). Learning environments impacted by shifting educational goals and demands, such as the incorporation of technology into the curriculum, are called innovative learning spaces (Khamitova, 2023). There exist diverse types of innovative learning spaces, such as technology-enhanced learning spaces, smart learning environments, hybrid learning spaces, active learning classrooms, learning studios, learning commons, and maker spaces, are becoming increasingly popular. These classroom settings are flexible, technological, and multimodal educational spaces that aim to meet the needs of 21st century learners (Benade, 2020; Dovey and Fisher, 2014; Mulcahy and Morrison, 2020; Tanner, 2008; Bannister, 2017).

These innovative classrooms are linked to a more student-centered, technology-mediated, which promotes personalized learning and the flexible application of a variety of learning approaches (Dovey and Fisher, 2014; Dumont and Istance, 2010). Therefore, eliminating spatial barriers such as rigid seating arrangements and facilitates the implementation of student-centered and collaborative strategies (Dumont and Istance, 2010; Mirici and Kızılışıkoğlu, 2025). One type of innovative classroom is the FCL that was promoted by European Schoolnet. It aims to prepare students to be citizens for the 21st century and equip them with the skills needed to find their place in a rapidly changing world. This requires new teaching scenarios and learning spaces that facilitate the incorporation of new pedagogical approaches and active learning (Baeta and Pedro, 2017; Dúo-Terrón, 2024; Garnier, 2016), where students work autonomously, acquiring and experiencing their own learning, not only acquiring knowledge but also competences (Martínez and Gómez, 2025). The FCL was founded based on the findings of the Innovative Technologies for an Engaging Classroom (iTEC) project, which was supported by the European Commission (Blahová et al., 2013; Nedel and Buzzar, 2020) and aimed to encourage a transition away from the TC to a more flexible learning environments and active methodologies-based labs (Jin and Peng, 2022; Pedro et al., 2017). This innovative learning space, FCL, is characterized by having six different learning areas (create, interact, present, investigate, exchange, and develop) and offers diverse technological tools (Colton et al., 2020; Montero-Izquierdo et al., 2025; Saari and Decuypere, 2024). The selection of the FCL as the learning classroom for mathematics education requires a methodological shift in the educational initiatives, providing a new pedagogy where interaction is the primary component to create a dynamic and flexible classroom environment (Qiu, 2014; Bannister, 2017). The learning scenarios proposed in the FCL are guided by teachers, which act as facilitators of students’ learning and allow learners to use digital tools and learning spaces (Hickman, 2016; Eken et al., 2026). The aim of the FCL is to help teachers design educative proposals from an active approach to engage students in projects to think critically and seek out different points of view allowing teachers to provide constant feedback while students work in their projects (Ntuli and Kyei-Blankson, 2016). Consequently, the FCL learning environment invites teachers to reconsider the use of active and innovative methodological practices, focusing on active and competency-based learning (Dúo-Terrón, 2024; Garnier, 2016). Therefore, the use of FCL in mathematics education aims to promote not only content knowledge, but also PSTs participation, experimentation, research, and interaction.

Although previous studies largely agree that versatile, active learning classrooms featuring flexible layouts and integrated technology promote student engagement, motivation, and collaboration (Lewohl, 2023), a persistent debate remains regarding whether these high-tech, multifunctional configurations induce cognitive overload or technostress among PSTs (Dinc, 2019; Yang et al., 2025). This specific theoretical tension establishes the physical baseline comparison between the TC and the flexible learning environment evaluated in the current study, allowing us to examine whether these active spaces act as pedagogical stressors or as affective catalysts.

### 2.2 Affective domain: emotion and self-efficacy belief

In this study, two variables of the affective domain, emotion and self-efficacy belief, have been the subject of research. These variables are interrelated; self-efficacy belief can be considered both a precursor and a consequence of emotions. According to several theories, there is a relationship between the influence of emotions and self-efficacy beliefs on commitment to learning. The control that students perceive over learning activities and the value they place on them are what motivate their feelings of achievement (Pekrun, 2006; Pekrun and Perry, 2014; Pekrun, 2024).

When positive emotions such as pleasure and satisfaction arise in students, this can further boost their academic engagement and enhance their creative abilities (Bergdahl et al., 2023; Chiu et al., 2024; Jin and Ye, 2022). Additionally, emotional reactions are influenced both by the degree of control students feel over learning tasks and by the value they place on them (Pekrun, 2006; Goetz et al., 2026), and self-efficacy belief increases students’ sense of control over activities, which in turn generates positive feelings such as interest and satisfaction (Joo et al., 2013; Pekrun and Linnenbrink-Garcia, 2012; Zhao and Liu, 2022).

On the other hand, self-efficacy belief is a key determinant of academic engagement, and earning behaviors are influenced by a combination of environmental and personal cognitive factors (Woreta et al., 2025; Olivier et al., 2019). Students may feel less anxious when they have high self-efficacy belief (Kim and Hodges, 2012; Pekrun et al., 2007). Therefore, students are more likely to have positive emotional reactions like interest and satisfaction when their confidence in learning activities is increased. According to Wang et al. (2022), this emotional state in turn encourages students to put in more time and effort as well as to persevere through and finish learning assignments. People are influenced by their self-efficacy beliefs, which also shape their coping skills and emotion regulation mechanisms, their determination to persevere in the face of adversity, their outcome expectations and causal attributions of successes and failures (Bandura, 2012). Self-efficacy belief in teaching is a key factor and can be defined as teachers’ confidence in their ability to instruct their subject matter, efficiently run the classroom, and inspire and engage students to learn (Tschannen-Moran and Hoy, 2001). PSTs and novice teachers can develop their self-efficacy beliefs by seeing mentors, who can offer vicarious experiences (Posnanski, 2002; Rice and Roychoudhury, 2003). Finally, self-efficacy belief is a crucial component in education that affects students’ academic performance and learning habits. Zimmerman (2000) showed that increasing students’ confidence and enthusiasm for learning tasks and self-efficacy belief dramatically improves their engagement and behavior to actively participate in academic activities. Additionally, Sökmen (2021) showed in research of college students that raising students’ self-efficacy belief might boost their academic engagement and, consequently, their academic achievement. While there is a clear consensus in the literature that positive emotions are closely correlated with higher levels of self-efficacy beliefs, there remains a lack of agreement regarding how quickly these affective states can change when the learning environment is modified; thus, some authors argue that affective traits are deeply stable, while others consider them to be situational (Hannula, 2019; Schukajlow et al., 2023). The present study directly addresses this ongoing debate by empirically testing whether a strategic change in the environment can dynamically improve these traditionally ingrained affective traits in mathematics teachers.

### 2.3 Impact of the FCL learning setting on PSTs’ emotion and self-efficacy belief in mathematics education

A large body of research has demonstrated a significant relationship between the perceived learning environment and emotional factors (Anderman, 2002; Anderman et al., 2001; Frenzel et al., 2007; Turner et al., 2002; Schukajlow et al., 2023). Many emotions are experienced by learners in different learning contexts (Frenzel et al., 2007). Consequently, the relationships between students’ perceptions of the classroom environment and their emotional experiences in mathematics education remain largely unknown. Nevertheless, it is crucial to recognize the importance of students’ emotional experiences as outcome variables, given their impact on students’ well-being and learning (Salovey et al., 2000). Empirical research has demonstrated that students’ performance levels in mathematics correlate with their emotions and experience in the subject, for instance negative emotions have been found in low-achieving students (Forsblom et al., 2022; Holm et al., 2017; Holm et al., 2020). Students are known to experience a variety of achievement emotions when studying mathematics (Frenzel et al., 2007). Especially, enjoyment, anger, and boredom were found to influence students’ performance in mathematics (Forsblom et al., 2022; Montero-Izquierdo et al., 2025).

Teacher self-efficacy belief in mathematics refers to the conviction that one can carry out mathematics teaching activities successfully. Therefore, studying teacher self-efficacy within the subject of mathematics education is crucial. In academic settings, students’ achievement emotions are linked to learners’ performance, and a variety of factors have a significant impact on students’ subjective control and subjective value, which in turn affects their achievement emotions (Pekrun, 2006). The environmental factors that have been linked to achievement emotions are the classroom setting, teachers’ feedback, student’s goals, teacher support, and interactions (Holm et al., 2017; Holm et al., 2020; Pekrun, 2006; Pekrun et al., 2006). Regarding the learning settings that PSTs perceived positively are related to greater self-efficacy belief in teaching mathematics (McMinn et al., 2021). Previous research has shown that instructors’ performance is influenced by their level of teaching self-efficacy belief (Duffin et al., 2012; Olawale and Hendricks, 2024; Orakcı et al., 2023).

The characteristics of most teachers who have higher levels of self-efficacy belief have positive teaching attitudes and use student-centered approaches in the classroom (Gresham, 2008; Hwang, 2021; Muijs and Reynolds, 2015). According to Peker (2016), self-efficacy beliefs have a crucial role in the affective domain of mathematics education. Many teachers have doubts about their ability in mathematics, and teacher self-efficacy belief is thought to be subject-specific (Charalambous et al., 2008; Solanon and San Jose, 2025).

The teaching methodology seemed to be influenced by space, which in turn affected learning experiences and, it was found that the importance of flexible spaces, supported by technology, had a positive significant effect on students’ performance in mathematics education (Imms and Byers, 2017). As aforementioned, the FCL is a flexible learning space that integrates the use of technology, student-centered approaches, and active learning methodologies, in which students actively construct knowledge. Some authors have measured emotion in technology-rich learning environments (Lajoie et al., 2020; Wang et al., 2023). Other authors studied the effect of active learning classrooms on student emotions, which resulted in an increase in students’ satisfaction, motivation to improve the learning experience, learning outcomes, and student performance (Berssanette and de Francisco, 2021; Kuwabara, 2024). Moreover, when constructivism approach was applied in the learning environment, students had higher self-efficacy belief in comparison to the traditional lecture-based environment (Alt, 2015). Some authors suggested that using an innovative learning classroom in conjunction with a suitable teaching strategy can boost students’ self-efficacy belief and learning engagement (Shi et al., 2018). In other study, it was research about the interrelationship among academic emotions, classroom engagement, and self-efficacy belief in the context of learning in an innovative learning space and the findings shown that has the potential to boost students’ self-efficacy belief by encouraging good academic feelings and increasing learners’ engagement in the learning process (Hu et al., 2024; Zhao and Liu, 2022). In concern with students’ emotions, the smart classroom setting contributes to fostering good feelings and reducing negative ones which encourage student participation, which eventually affects the students’ educational experiences and academic achievement (Ma et al., 2024). As is apparent from the existing literature, only a single study has analyzed the learning space in mathematics learnings. All the others share certain characteristics of the FCL, including active learning through active methodologies, a constructivist approach, and its effect on students’ emotions or self-efficacy beliefs. However, neither of them refers to the FCL, nor to mathematics education. Therefore, this study aims to address this research gap.

## 3 Methods and materials

### 3.1 Participants

The sample comprised 105 PSTs, of whom 54 were assigned to the TC group and 51 to the FCL group. At the time of the study, all participants were second-year students enrolled in Mathematics and its Didactics subject, a course offered during the second semester of the bachelor’s degree program in Primary Education. The intervention was conducted at the public university with which the authors are affiliated. A quasi-experimental design involving two intact class groups was used because individual random assignment was not feasible owing to scheduling and organizational constraints. The same instructor taught all sections, following the official curriculum of the authors’ university; consequently, both cohorts received identical instructional time, curricular content, and assessment structures. Groups homogeneity was assessed using scores from a pre-content test, which indicated comparable performance between groups. Finally, Table 1 summarizes the participant sample demographic characteristics. In the TC group, 54% of PSTs were female and 46% male; in the FCL group, 59% were female and 41% male. Regarding prior academic background, in the TC group 70% had a social sciences background, 24% science, 4% technology, and 2% other; in the FCL group, 73% had a social sciences background, 25% science, and 2% technology.

**Table 1:** Demographic information on the study sample for Cohort 1 (Traditional Classroom, TC) and Cohort 2 (Future Classroom Lab, FCL).

Learning environment	Cohort 1	Cohort 2
	TC	FCL
*N*	54	51
Age	20.30	20.31
Educational background	%	
Social Science	70	73
Science	24	25
Technology	4	2
Others	2	—
Gender	%	
Female	54	59
Male	46	41

### 3.2 The classroom settings spaces: TC and FCL

The research was conducted in two different learning settings: a TC and FCL as shown in Figure 1. Regarding the distribution of the TC, the chairs and desks are positioned in fixed rows. The teacher’s desk, the chalkboard, the data projector at the front of the room, and the desks were oriented toward the teaching position. This frontal spatial layout highlights a didactic approach that is teacher centered. By contrast, the FCL was organized into multiple learning zones and integrated educational technologies. It included a display screen and projector, robust Wi-Fi to support device connectivity, and mobile furniture to enable rapid reconfiguration of the space according to the instructional method employed.

**Figure 1:**
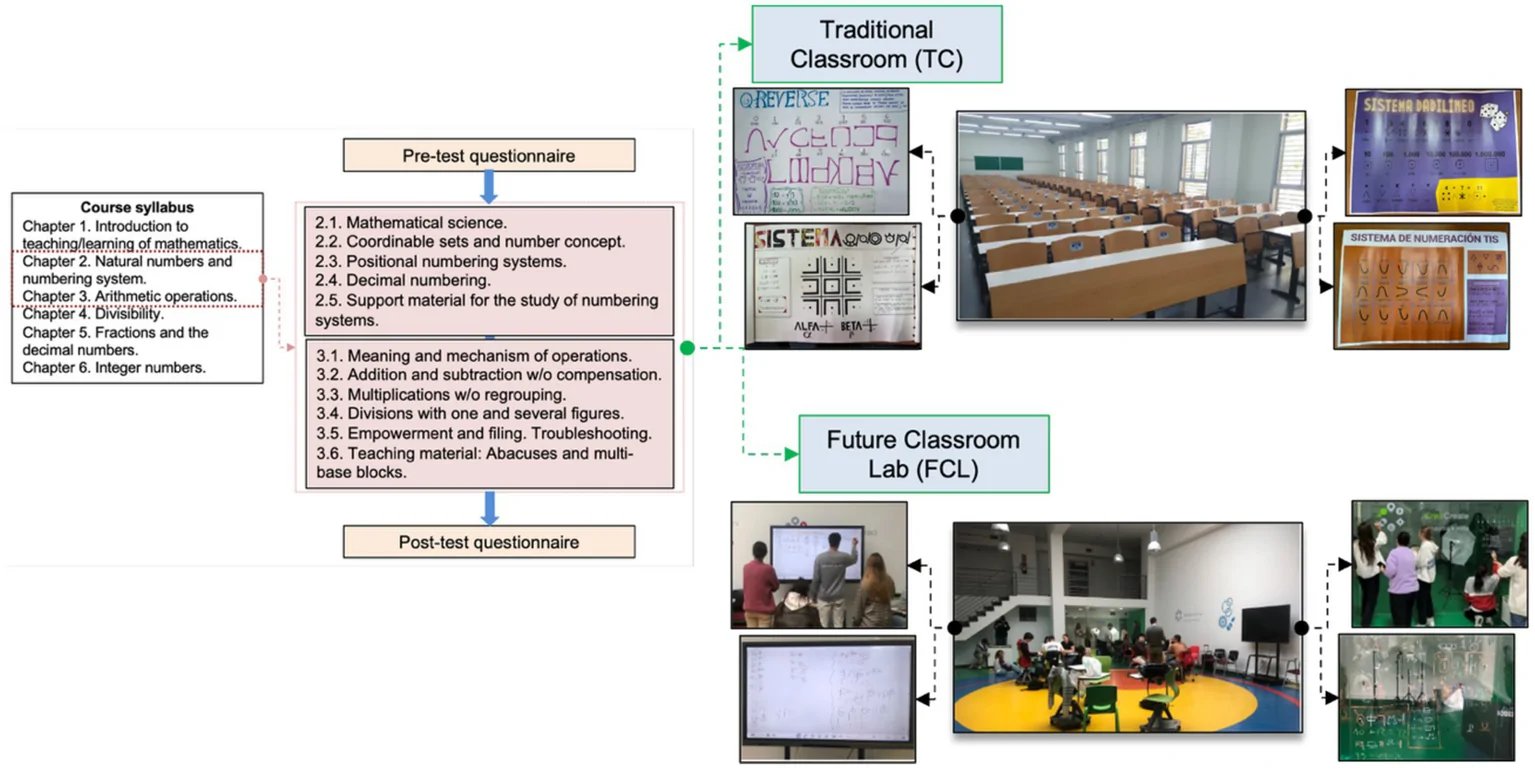
Different learning settings: TC and FCL.

At the time of the intervention, the instructors had 4 years of experience teaching Mathematics and its Didactics using learning activities designed for a Future Classroom Lab environment. The FCL was established at the authors’ university to provide PSTs with opportunities to experience and become familiar with innovative learning environments that they could potentially use in their future teaching practice. The participants were pre-service primary education teachers, whereas the intervention was designed and delivered by experienced university instructors.

The intervention provided the participants with an opportunity to apply theoretical contents that had previously been taught in traditional classroom sessions. The technological devices available in the FCL were used only during this specific session of the course. This session was the participants’ first experience of learning in an FCL environment. The experience also enabled the instructors to reflect on the implementation of the activity and identify potential improvements for its future design.

### 3.3 Procedure

The study was embedded within Mathematics and its Didactics, a 60-h course delivered over 15 weeks, with 4 hours of instruction per week. The intervention was implemented throughout Units 2 and 3 of the course syllabus, titled “Natural Numbers and Number Systems” and “Arithmetic Operations” as shown in Figure 1. Together, these units comprised 24-h of instruction. The wrap-up activity described below constituted the culminating activity of this 24-h instructional sequence.

During this culminating activity, students were asked to design a numeral system and perform arithmetic operations within that system. The activity enabled them to apply the mathematical contents, teaching strategies, and instructional resources introduced throughout Units 2 and 3. The same instructor taught both groups in accordance with the university’s official curriculum. Consequently, both groups received the same instructional time, curricular content, learning objectives, and assessment procedures. The study compared the outcomes associated with implementing the same instructional sequence in two different learning environments: the TC and the FCL.

The PSTs were organized into medium-sized groups and collaboratively devised a numeral system capable of coherently representing any whole number. To achieve this, they followed the grouping principle, which involves introducing a new symbol to represent higher-order units formed by grouping lower-order units. They then developed a set of symbols for their numeral system and explained its correspondence with the decimal system. Finally, the students performed addition, subtraction, multiplication, and division using their newly developed system.

In the TC condition, students used cardboard to model their numeral systems and presented their results orally to the class. In the FCL condition, some students designed their systems using the interactive digital display, whereas others used the writable glass wall. The students subsequently produced a video presentation in a chroma-key studio to explain their numeral systems and results. Figure 2 presents a flowchart of the intervention procedure.

**Figure 2:**
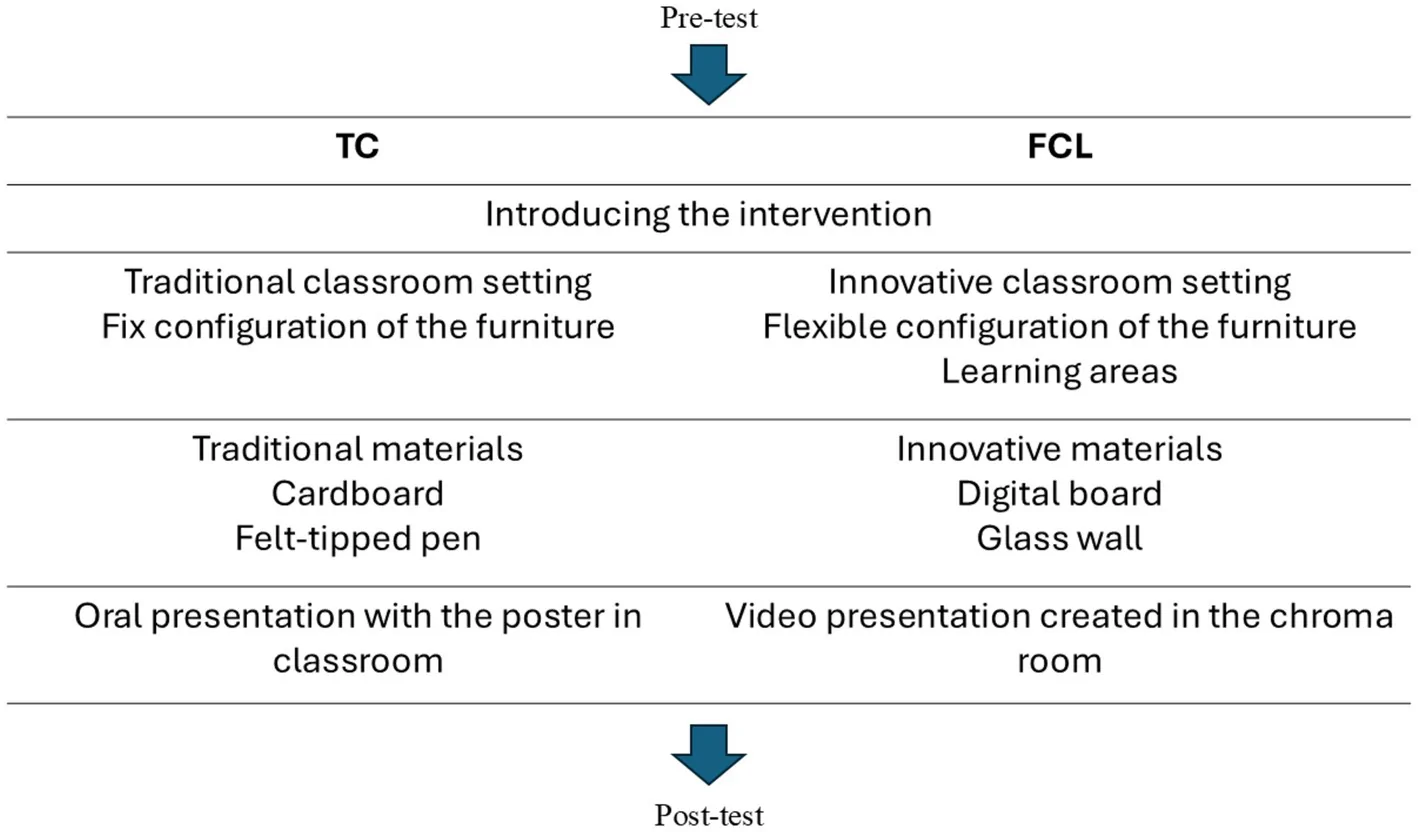
Flowchart of the intervention procedure in the different learning settings: TC and FCL.

### 3.4 Instrument

The instrument was administered online before and after the intervention (pre- and post-test) to collect participants’ responses. The instrument was based on a 5-point Likert-type questionnaire was used to measure the dependent variables (emotions and self-efficacy beliefs). The questionnaire comprised 23 items: 14 assessing emotions and 9 assessing self-efficacy beliefs in mathematics learning (see Table 2). The emotions scale was adapted from a previously established instrument by Pekrun et al. (2011), which had been adapted to this context in prior work (Jeong and González-Gómez, 2026). The items used to measure self-efficacy beliefs were adapted from the Science Teaching Efficacy Belief Instrument (STEBI-B) for the mathematics context (Jeong et al., 2019). The emotions questionnaire was not modified; rather, a specific subset of items was selected to align with the framework of a broader research project. This selection was strictly maintained across phases to ensure methodological homogeneity and to enable a direct, reliable comparison between the different studies within the project. The self-efficacy items were adapted to mathematics contents. Moreover, the study questionnaire was reviewed and approved by the Bioethics and Biosafety Committee at the authors’ university (registration nos. 94/2018, 77/2023, and 200/2024). Participants received written information at the start of the online questionnaire. Informed consent guaranteed anonymity and confidentiality and stated that the data would be used exclusively for research purposes.

**Table 2:** The questionnaire instrument and items.

Questionnaires	
1. Emotions items	2. Self-efficacy beliefs items
Positive emotions EM1. Joy EM2. Satisfaction EM3. Enthusiasm EM4. Fun EM5. Confidence EM6. Hope EM7. Pride Negative emotions EM8. Uncertainty EM9. Nervousness EM10. Concern EM11. Frustration EM12. Boredom EM13. Fear EM14. Anxiety	SE1. I understand mathematics concepts well enough to teach mathematics at lower educational levels. SE2. I will usually be able to answer students’ mathematics questions. SE3. When I put all my efforts, I will succeed in teaching mathematics as well as I would in other subjects. SE4. I believe I have the necessary skills to teach mathematics. SE5. Mathematics is useful for solving everyday problems. SE6. It is important to know mathematics to get a good job. SE7. I know the steps necessary to teach mathematics effectively. SE8. I encounter difficulties when trying to explain a mathematical concept. SE9. The use of motivating teaching spaces is essential to achieve good learning results.

### 3.5 Statistical analyses

Before analyzing the influence of learning settings on emotions and self-efficacy beliefs felt by the PSTs, a three-factor Confirmatory Factor Analysis (CFA) was estimated with positive emotions, negative emotions, and self-efficacy beliefs in mathematics using WLSMV (DWLS; mean-adjusted, scaled-and-shifted). Global fit was very good, χ^2^(246) = 451, *p* < 0.001, CFI = 0.992, TLI = 0.992, RMSEA = 0.068 (90% CI [0.058, 0.077], close-fit test *p* = 0.002), and SRMR = 0.070; a scaled/robust SRMR was also provided (0.061). Standardized factor loadings were uniformly strong: Positive Emotions (EMP) βs ranged 0.745–0.840, Negative Emotions (EMN) βs ranged 0.458–0.830, and Self-Efficacy beliefs (SE) βs ranged 0.695–0.915. Latent factor correlations were large in theoretically consistent directions suggesting close relations among constructs; thresholds for all indicators were estimated under an ordered-categorical specification. The instruments demonstrated robust internal consistency across all constructs and for both groups, with Cronbach’s alpha coefficients ranging from 0.828 to 0.861 for TC (PE = 0.861, NE = 0.857, SE = 0.828) and from 0.764 to 0.914 for FCL (PE = 0.910, NE = 0.764, SE = 0.914), all well exceeding the acceptable psychometric threshold of 0.70 (see Table 3). Based on these results, each construct global score (G-EMP, G-EMN, and G-SE) was computed as the equally weighted the scores of all items comprising that construct. To assess the influence of two educational environments (TC vs. FCL) on the construct global score an analysis of variance (ANOVA) was conducted before and after the intervention in the two different learning settings. When significant differences were found, the effect size was measured (η2). Additionally, a post-hoc test was performed to identify significant differences between groups. When the post-hoc analysis revealed the existence of significant differences, the effect size was measured using Cohen’s d. Finally, ANCOVA test was used to determine the possible effects of the gender and background on the emotions and self-efficacy beliefs felt by the PSTs based on the learning environment.

**Table 3:** Questionnaire constructs and Cronbach’s alpha coefficient (α) for the two groups analyzed: TC and FCL.

Questionnaire constructs	Cronbach’s alpha coefficient (α)	
	TC	FCL
Positive emotions	0.861	0.910
Negative emotions	0.857	0.764
Self-efficacy beliefs	0.828	0.914

To examine distributional assumptions, the Shapiro–Wilk test was applied for both study groups, indicating non-normality. Consequently, nonparametric statistical tests were used. A Mann–Whitney test was conducted to examine differences in the variables before and after the intervention. Median differences were evaluated using the Mann–Whitney test, and effect sizes were computed using the rank-biserial correlation (*rrb*) for nonparametric data in cases where significant differences were found (Cureton, 1968; Kerby, 2014). Statistical analyses were performed using Jamovi (version 2.4.11) and JASP (version 0.19.3).

Finally, Spearman’s rank correlation was used to examine positive and negative associations between emotions and self-efficacy beliefs in mathematics for both learning settings (TC and FCL groups). Spearman’s rho (*ρ*) was obtained, and correlations were considered high when *ρ* > 0.60.

## 4 Results

This study examined the effects of two instructional learning settings, TC and FCL, on PSTs’ emotions and self-efficacy beliefs, factors closely linked to students’ learning, motivation, and performance in mathematics education.

To assess the influence of the instructional environment, pre- and post-intervention mean values for the global positive emotions (G-EMP), negative emotions (G-EMN), and self-efficacy beliefs (G-SE) in mathematics were compared using ANOVA. Assumptions of normality were met. A significant effect of the intervention on PSTs’ global positive emotions (G-EMP) was indicated, *F*(3, 206) = 167.00, *ρ* < 0.001, η^2^ = 0.709, representing a large effect of the instructional environment on PSTs’ positive emotions. Significant pre–post increases in G-EMP were identified in both environments (Holm-adjusted *ρ* < 0.005): ΔM = 1.35 for TC and ΔM = 1.46 for FCL, with a larger improvement observed in the FCL group. Similar results were obtained for global negative emotions (G-EMN): a significant effect of the intervention was found, *F*(3, 206) = 209.00, *ρ* < 0.001, η^2^ = 0.753, reflecting a large effect of the instructional environment on PSTs’ negative emotions. Significant pre–post decreases in G-EMN were observed (Holm-adjusted *p* < 0.005): ΔM = −1.22 for TC and ΔM = −1.39 for FCL, with a larger decrease observed in the FCL group. Finally, for global self-efficacy beliefs (G-SE), a significant intervention effect was observed, *F*(3, 206) = 378.00, *p* < 0.001, η^2^ = 0.846, indicating a large effect of the instructional environment on PSTs’ self-efficacy beliefs. Significant pre–post increases in G-SE were found (Holm-adjusted *ρ* < 0.005): ΔM = 1.27 for TC and ΔM = 2.13 for FCL, with a larger increase observed in the FCL group. Finally, the ANCOVA results indicated that no significant influences were observed when considering gender or educational background for G-EMP, G-EMN and G-SE (*ρ* > 0.05 in all cases).

Once global constructs were analyzed, a Mann–Whitney test was conducted to examine differences in the variables before and after the intervention in the two different learning settings (TC and FLC). Table 4 reports the *p*-values for the emotions and self-efficacy beliefs measures.

**Table 4:** Median differences in the emotions and self-efficacy beliefs items comparing pre- and post-test for TC and FCL learning settings.

Learning setting	Cohort 1				Cohort 2			
	TC				FCL			
Items	Mdn Pre-test	Mdn Post-test	*p*	rbb	Mdn Pre-test	Mdn Post-test	*p*	rbb
EM1	4	5	<0.001	0.717	3	5	<0.001	0.842
EM2	4	5	<0.001	0.628	3	5	<0.001	0.826
EM3	4	5	<0.001	0.727	3	5	<0.001	0.571
EM4	3	5	<0.001	0.763	3	5	<0.001	0.751
EM5	3	5	<0.001	0.879	3	5	<0.001	0.787
EM6	3	5	<0.001	0.954	3	5	<0.001	0.542
EM7	4	5	<0.001	0.678	3	5	<0.001	0.668
EM8	3	1	<0.001	0.947	4	2	<0.001	0.825
EM9	3	1	<0.001	0.805	2	1	<0.001	0.562
EM10	2	1	<0.001	0.588	3	1	<0.001	0.612
EM11	2	1	<0.001	0.369	3	3	0.169	0.152
EM12	2	1	<0.001	0.707	4	3	0.003	0.328
EM13	3	1	<0.001	0.746	4	1	<0.001	0.804
EM14	3	2	<0.001	0.861	4	1	<0.001	0.749
SE1	4	5	0.001	0.324	1	4	<0.001	0.913
SE2	4	5	<0.001	0.414	2	4	<0.001	0.876
SE3	3	4	<0.001	0.559	2	5	<0.001	0.670
SE4	3	5	<0.001	0.599	2	4	<0.001	0.722
SE5	4	5	<0.001	0.362	2	5	<0.001	0.802
SE6	4	4	<0.001	0.466	2	4	<0.001	0.895
SE7	3	4	<0.001	0.588	3	4	<0.001	0.622
SE8	2	1	0.003	0.306	4	2	<0.001	0.855
SE9	3	4	<0.001	0.567	2	5	<0.001	0.832

Across the 23 items assessing emotions and self-efficacy beliefs, most pre–post comparisons were statistically significant (*ρ* < 0.05), and many exhibited very large effect sizes (*rbb* ≥ 0.80; see Figure 3). In the TC setting, five items showed very large effect sizes (EM5, EM6, EM8, EM9, and EM14). Conversely, eight items exhibited effects of this magnitude within the FCL setting (EM1, EM2, SE1, SE2, SE5, SE6, SE8, and SE9), demonstrating that a higher number of prominent effects occurred in the FCL compared to the TC. In the TC setting, increases were most pronounced for confidence and hope (EM5 and EM6), whereas in the FCL setting the largest effects were observed for joy and satisfaction (EM1 and EM2). For negative-emotion items, all were significant in TC; in FCL, frustration (EM11) did not change significantly from pre to post. Regarding self-efficacy beliefs items, very large effects were observed for SE1, SE2, SE5, SE6, SE8, and SE9 in FCL. Taken together, these results indicate that the FCL environment produced larger and more numerous item-level effects than the TC environment in the context of mathematics instruction.

**Figure 3:**
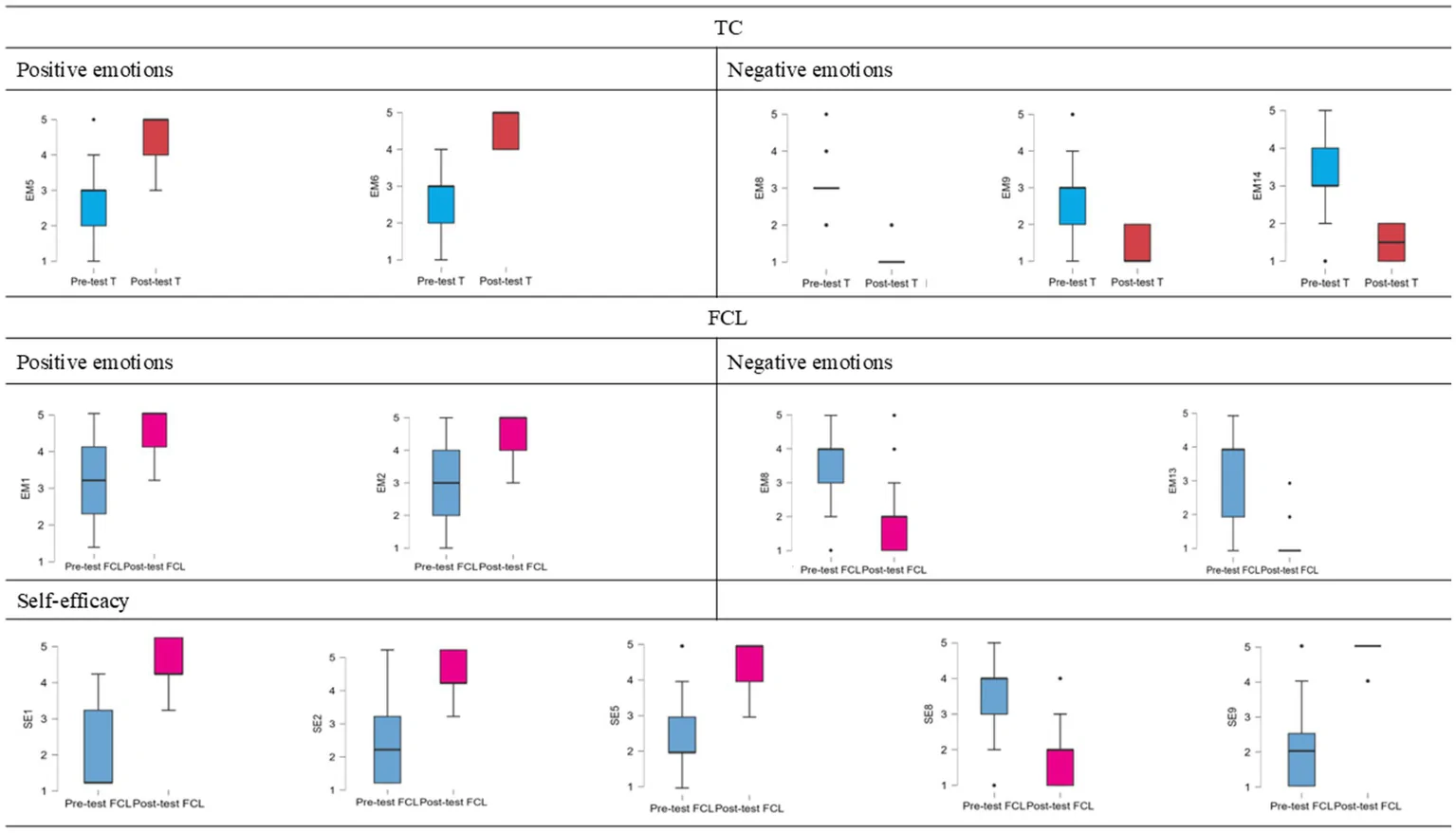
Significance of median differences in the items analyzed in TC in comparison to FCL learning settings.

### 4.1 Correlations of emotions and self-efficacy beliefs in TC and FCL learning settings

The correlations between PSTs’ emotions and self-efficacy beliefs were analyzed across the TC and FCL settings within the mathematics intervention (see Table 5). Overall, 76.2% of the stronger correlations were observed in the FCL setting, whereas 23.8% were observed in the TC. In the FCL group, all positive emotion items correlated significantly with six self-efficacy belief items (SE1, SE2, SE5, SE6, SE8, and SE9). Conversely, in the TC group, positive emotion items correlated more strongly with the remaining three self-efficacy belief items (SE3, SE4, and SE7). Therefore, as summarized in Table 5, the associations between positive affect and teaching confidence in mathematics were generally more robust in the FCL setting than in the TC. These findings reveal a strong positive association between pre-service teachers’ emotional states and their self-efficacy beliefs within the TC setting, indicating that favorable emotions closely align with teaching confidence.

**Table 5:** Correlations between positive emotions and self-efficacy beliefs in TC and FCL learning settings.

Variable	Classroom	EM1 Joy	EM2 Satisfaction	EM3 Enthusiasm	EM4 Fun	EM5 Confidence	EM6 Hope	EM7 Pride
SE1	TC	0.442	0.317	0.277	0.273	0.312	0.348	0.216
	FCL	0.677	0.548	0.426	0.606	0.565	0.488	0.439
SE2	TC	0.693	0.362	0.436	0.336	0.417	0.382	0.333
	FCL	0.662	0.552	0.494	0.552	0.598	0.445	0.465
SE3	TC	0.393	0.370	0.675	0.519	0.541	0.474	0.436
	FCL	0.473	0.583	0.451	0.517	0.535	0.463	0.488
SE4	TC	0.354	0.408	0.594	0.633	0.568	0.493	0.335
	FCL	0.543	0.530	0.425	0.612	0.508	0.450	0.567
SE5	TC	0.344	0.404	0.247	0.325	0.363	0.405	0.193
	FCL	0.499	0.569	0.508	0.605	0.615	0.480	0.465
SE6	TC	0.304	0.461	0.357	0.356	0.378	0.499	0.345
	FCL	0.662	0.566	0.501	0.614	0.641	0.512	0.575
SE7	TC	0.456	0.495	0.483	0.455	0.463	0.553	0.409
	FCL	0.432	0.381	0.299	0.535	0.528	0.460	0.362
SE8	TC	−0.111	−0.165	−0.354	−0.219	−0.357	−0.308	−0.254
	FCL	−0.580	−0.539	−0.465	−0.550	−0.598	−0.446	−0.560
SE9	TC	0.401	0.584	0.439	0.435	0.509	0.444	0.386
	FCL	0.666	0.606	0.473	0.481	0.518	0.358	0.438

Table 6 presents the correlations between negative emotions and self-efficacy beliefs for the TC and FCL learning settings. When correlations were compared across settings, 60.32% of the stronger correlations were observed in the FCL, whereas 39.68% were observed in the TC. Specifically, in the FCL, negative emotions correlated more strongly with seven self-efficacy beliefs items (SE1, SE2, SE4, SE5, SE6, SE8, and SE9); conversely, in the TC, stronger correlations with negative emotions were observed for the remaining two items (SE3 and SE7). Accordingly, a greater number of strong correlations between negative emotions and self-efficacy beliefs in mathematics items were observed in the FCL than in the TC. Therefore, within the FCL, greater self-efficacy beliefs were associated with a lower prevalence of negative emotions.

**Table 6:** Correlations between negative emotions and self-efficacy beliefs in TC and FCL learning settings.

Variable	Classroom	EM8 Uncertainty	EM9 Nervousness	EM10 Concern	EM11 Frustration	EM12 Boredom	EM13 Fear	EM14 Anxiety
SE1	TC	−0.334	−0.300	−0.340	−0.188	−0.329	−0.326	−0.337
	FCL	−0.653	−0.467	−0.479	−0.062	−0.190	−0.648	−0.685
SE2	TC	−0.365	−0.311	−0.298	−0.259	−0.348	−0.378	−0.399
	FCL	−0.587	−0.438	−0.498	−0.033	−0.256	−0.657	−0.543
SE3	TC	−0.435	−0.428	−0.340	−0.259	−0.403	−0.484	−0.464
	FCL	−0.443	−0.291	−0.434	−0.119	−0.167	−0.396	−0.530
SE4	TC	−0.448	−0.345	−0.414	−0.266	−0.398	−0.483	−0.432
	FCL	−0.517	−0.395	−0.427	−0.104	−0.274	−0.422	−0.469
SE5	TC	−0.348	−0.337	−0.249	−0.374	−0.334	−0.304	−0.344
	FCL	−0.526	−0.382	−0.436	−0.140	−0.203	−0.603	−0.604
SE6	TC	−0.489	−0.433	−0.366	−0.507	−0.326	−0.469	−0.379
	FCL	−0.528	−0.474	−0.501	−0.140	−0.275	−0.648	−0.505
SE7	TC	−0.567	−0.455	−0.492	−0.434	−0.448	−0.451	−0.512
	FCL	−0.470	−0.232	−0.382	−0.091	−0.260	−0.377	−0.538
SE8	TC	0.262	0.306	0.278	0.187	0.302	0.403	0.318
	FCL	0.631	0.479	0.454	0.120	0.221	0.596	0.551
SE9	TC	−0.500	−0.346	−0.549	−0.451	−0.451	−0.484	−0.384
	FCL	−0.586	−0.474	−0.437	−0.188	−0.191	−0.663	−0.538

In Figures 4, 5, the correlations are displayed via the Spearman’s *rho* heatmap of the TC and FCL settings, respectively. The strongest correlations are highlighted in dark violet for positive correlation, and in dark red for negative correlation. Consequently, the findings revealed the strongest correlations (*rho* > 0.6) between positive emotions and self-efficacy belief with the TC setting, with a total of three correlations: (1) EM1-SE2, (2) EM3-SE3, and (3) EM4-SE4. Regarding the relationship between the measured variables, the TC setting showed an absence of strong associations (*rho* > 0.60) between negative emotions and self-efficacy belief. Conversely, the FCL environment dynamically intensified these affective links. Within the FCL, a substantial matrix of prominent positive correlations emerged, alongside multiple strong associations between negative emotions and self-efficacy belief items. This systemic contrast indicates that the flexible learning environment acts as an affective catalyst, making future teachers’ emotional states and their teaching confidence significantly more interconnected than in a traditional classroom setup.

**Figure 4:**
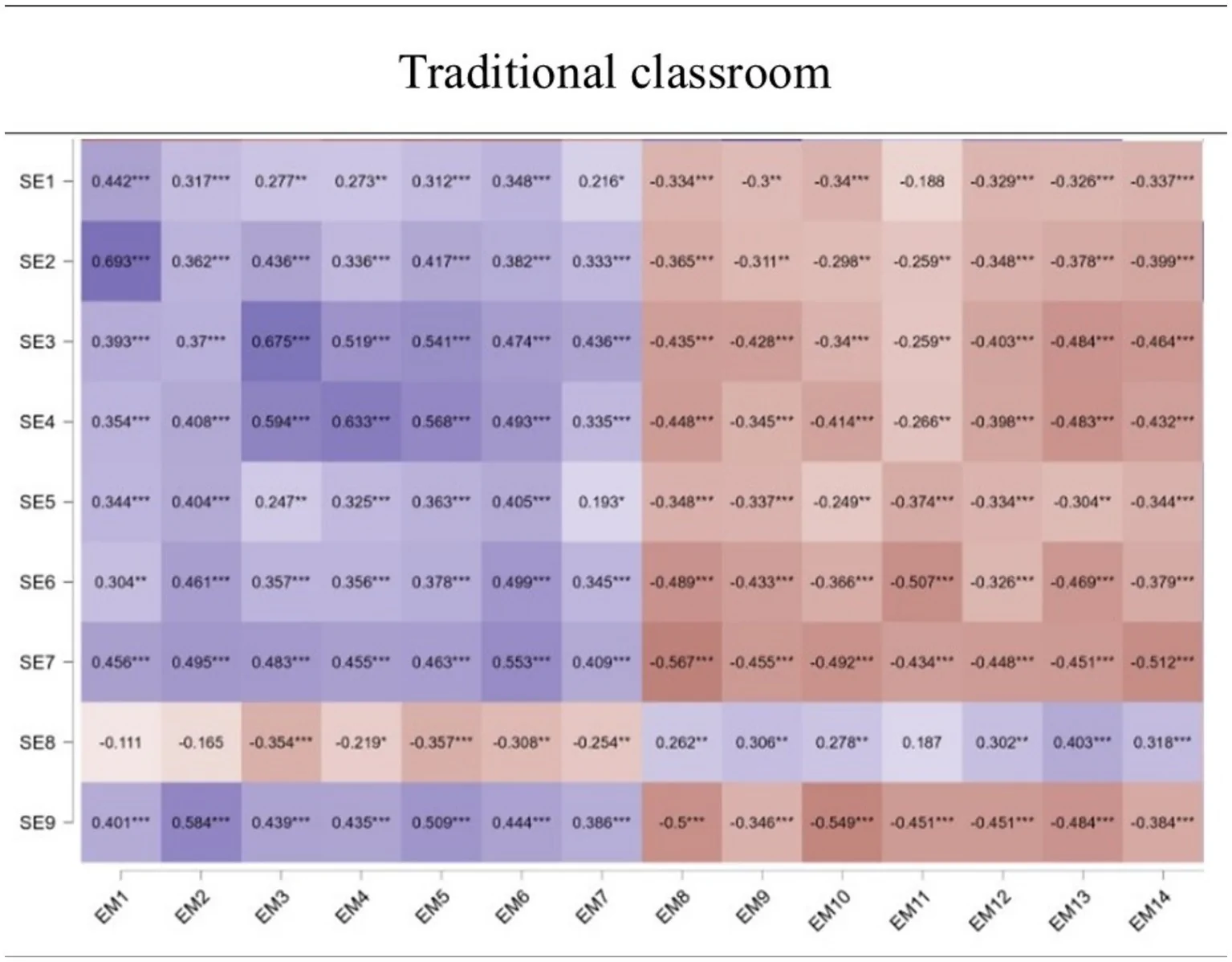
Correlation map Spearman’s *rho* heatmap of emotions and self-efficacy beliefs at the TC.

**Figure 5:**
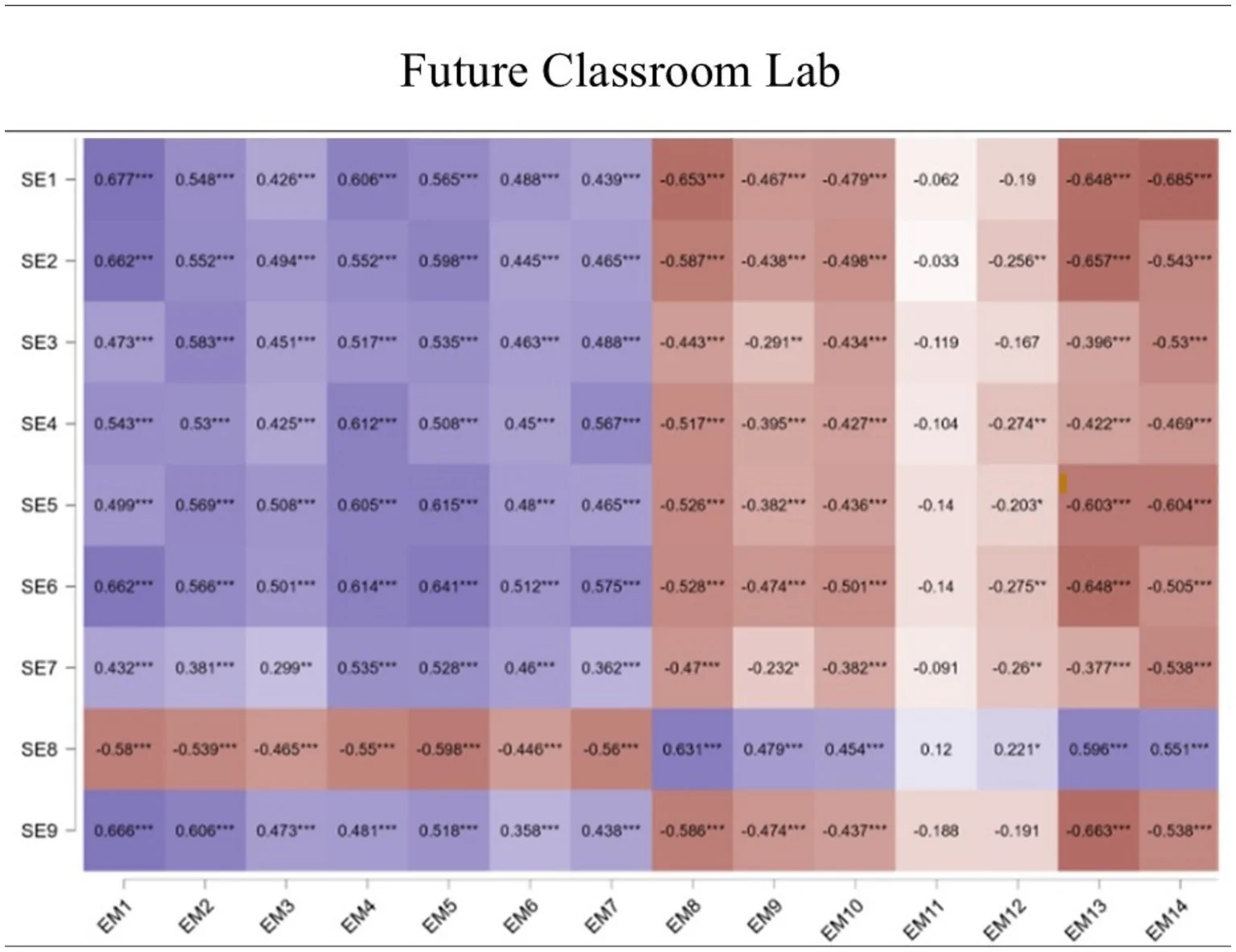
Correlation map Spearman’s *rho* heatmap of the emotions and self-efficacy beliefs at the FCL.

Overall, stronger correlations between emotions and self-efficacy beliefs were observed in the FCL than in the TC. These results suggest that flexible, technology-enhanced environments function as dynamic pedagogical catalysts, strengthening the positive reciprocal relationship between PSTs’ emotional states and their teaching confidence (Schukajlow et al., 2023).

## 5 Discussion

The emergence of innovative learning spaces has prompted this study to investigate whether their impact differs from that of TC and FCL settings in mathematics education. In particular, the FCL was selected as the analytical focus to address the empirical gap regarding its influence on PST emotions and self-efficacy beliefs, two affective domains construct whose contextual interactions remain under-researched in this field. Consequently, the present study aims to assess, through comparative analysis, whether the proposed intervention yields differential outcomes in learners’ emotions and self-efficacy beliefs of mathematics learnings depending on the learning setting where the intervention is applied. Therefore, this discussion clarifies the implications of the findings regarding how the three initial research questions were addressed through the study.

Regarding the first research question (RQ1), the FCL learning setting elicited significantly larger effects on PSTs’ emotions compared to the TC setting. While the TC environment primarily reinforced passive positive emotions, such as trust and hope, the FCL triggered highly arousing positive emotions, specifically joy and satisfaction, resulting in notably higher overall effect sizes (G-EMP and G-EMN). This divergence in emotional profiles is consistent with Pekrun’s (2006) Theory of Control and the Value of Achievement Emotions, which posits that learning environments that promote student autonomy and collaborative problem-solving directly enhance students’ positive emotions. These results indicate that different learning settings can elicit distinct emotional responses among PSTs and underscore the importance of examining emotions across different learning settings. The notable increase in joy and satisfaction in the FCL clearly reflects the empirical findings of Li et al. (2020) and Frenzel et al. (2007), who demonstrated that student-centered classroom environments alter emotional states through students’ active participation. Analyzing emotion is crucial in learning, as there is a well-documented connection between students’ emotion and the perception of the classroom environment (Frenzel et al., 2007; Li et al., 2020). In the FCL, activities are designed so that students work independently, collaborate, and engage in problem solving. Prior research indicates that independent work and problem solving can foster positive emotions (Pekrun, 2006), cooperative learning is positively associated with positive affect (Nummenmaa and Nummenmaa, 2008), and teaching practices that deliberately evoke positive emotions are effective (Hertel and Parks, 2002). In mathematics learning specifically, emotions play a pivotal role because they substantially shape learners’ motivation and academic achievement (Schoenherr et al., 2025). Therefore, implementing a FCL environment to elicit positive emotions may support teaching and learning in mathematics education.

Regarding the second research question (RQ2), the effect of TC and FCL learning settings on PSTs’ self-efficacy beliefs in mathematics were assessed. Self-efficacy beliefs is a key educational factor that influences students’ learning and academic achievement, and positive self-efficacy beliefs in mathematics have been linked to the classroom environment (Gilbert et al., 2014). Again, the assessment of the G-SE results show that a significant effect was observed, being this effect of higher magnitude in the FCL learning setting. Across the nine items of self-efficacy belief analyzed, five items (SE1, SE2, SE5, SE6, and SE8) showed small effect sizes in the TC condition, whereas the FCL condition exhibited very large effect sizes for the same items. Thus, the FCL environment produced larger effects on self-efficacy beliefs overall. Moreover, learning environments that PSTs perceive positively are associated with higher self-efficacy beliefs for teaching mathematics (McMinn et al., 2021). Fostering PSTs’ self-efficacy beliefs is essential for teaching because it reflects teachers perceived ability and confidence to teach effectively and to inspire and engage students (Tschannen-Moran and Hoy, 2001). Providing PSTs with innovative experiences can also serve as vicarious experiences that build self-efficacy beliefs (Posnanski, 2002; Rice and Roychoudhury, 2003). Furthermore, teachers with higher levels of self-efficacy exhibit significantly more positive teaching attitudes and are much more likely to use student-centered approaches in the classroom (Gresham, 2008; Hwang, 2021; Muijs and Reynolds, 2015). Therefore, the FCL intervention may help PSTs enhance their self-efficacy beliefs, with positive downstream effects on mathematics teaching practice and student engagement.

The third research question (RQ3) examined which learning setting, TC or FCL, shows stronger correlations between PSTs’ emotions and self-efficacy beliefs in mathematics education. Emotions influence self-efficacy beliefs, and positive emotions can enhance those beliefs (Medrano et al., 2016; Schutte, 2014). The findings revealed that correlations between positive emotions and self-efficacy beliefs were stronger in the FCL setting (76.2%) than in the TC (23.8%). In addition, higher self-efficacy beliefs are associated with a greater sense of control over activities, which in turn promotes positive feelings such as interest and satisfaction (Joo et al., 2013; Pekrun and Linnenbrink-Garcia, 2012). Regarding negative emotions, correlations with self-efficacy beliefs were also stronger in the FCL group (60.32%) than in the TC group (39.68%). Overall, the correlations between PSTs’ emotions and self-efficacy beliefs in mathematics were stronger when the intervention was implemented in the FCL environment. Students with high self-efficacy beliefs tend to report lower anxiety (Kim and Hodges, 2012; Pekrun et al., 2007). Specifically, in the FCL, all positive emotions were correlated with six of the total nine self-efficacy beliefs items (SE1, SE2, SE5, SE6, SE8, and SE9), whereas on the TC, positive emotions were more strongly correlated with the remaining three items (SE3, SE4, and SE7).

Therefore, when students feel more confident in learning activities, they are more likely to experience positive emotional reactions like interest and satisfaction. This emotional condition in turn motivates students to work more and persevere through learning assignments (Wang et al., 2022). The application of pedagogical proposals in the FCL can effectively promote positive PSTs’ emotions and self-efficacy beliefs, which reveals a positive effect of applying educational intervention for mathematics education in the FCL learning environment. Empirical research has demonstrated that students’ performance levels in mathematics correlate with their emotions and experience in the subject, for instance positive emotions have been found in high-achieving students (Forsblom et al., 2022; Holm et al., 2017; Holm et al., 2020). Also, the significant effects on reducing negative emotions were revealed in the results which have an impact on enhancing self-efficacy belief in mathematics. Finally, emotions and self-efficacy beliefs are correlated, and these beliefs are a fundamental component of education that affects students’ academic performance and learning habits. Therefore, fostering students’ confidence and enthusiasm for learning tasks and self-efficacy beliefs may dramatically improve their engagement and behavior to actively participate in academic activities (Zimmerman, 2000).

## 6 Conclusions, implications, and limitations

This study provides evidence that participation in a specific FCL-based mathematics activity was associated with more favorable immediate affective outcomes among PSTs. Compared with participants in the TC condition, those in the FCL condition reported lower levels of emotional rejection and stronger teaching self-efficacy beliefs following the intervention. These findings suggest that the flexible and technology-enhanced characteristics of the FCL environment may contribute to PSTs’ confidence and emotional engagement during mathematics education activities. However, given the specific context and duration of the intervention, the findings should not be interpreted as evidence of the long-term effectiveness or broader educational impact of the FCL approach.

The findings have potential implications for teacher education programs. University instructors may consider incorporating carefully designed activities in flexible, technology-enhanced environments into mathematics education courses. Such activities may provide PSTs with opportunities to engage actively with mathematical contents, apply previously acquired theoretical knowledge, and experience instructional approaches that they may consider using in their future teaching practice. Nevertheless, further research is required before conclusions can be drawn regarding whether these experiences influence PSTs’ subsequent classroom practices or produce sustained changes in their affective responses to mathematics.

An additional direction for future research would be to examine the implementation of FCL-based activities in inclusive and Special Education settings, including Integrated Co-Teaching and self-contained classrooms. The flexible organization of FCL environments may provide opportunities for PSTs to experience collaborative instructional practices and consider how learning spaces and activities can be adapted to diverse educational needs. However, these contexts were not examined in the present study; therefore, no conclusions can be drawn regarding the applicability or effectiveness of the FCL approach in such settings. Future research should evaluate its implementation with different student populations, instructional models, and educational contexts.

Despite the positive outcomes, several limitations must be acknowledged. First, the sample was drawn from a single institutional context, which may restrict the generalizability of the findings to broader or more culturally diverse populations of PSTs. In addition, data on participants’ socioeconomic backgrounds, prior academic performance, and specific learning levels were not collected, limiting the assessment of group comparability and the extent to which the findings can be generalized. Second, this study relied primarily on self-reported questionnaires to measure emotional factors and self-efficacy beliefs; incorporating qualitative methods, such as classroom observations or semi-structured interviews, could provide a more nuanced understanding of the factors that can affect these changes. Future research should consider longitudinal designs to examine whether these positive affective shifts are sustained during actual teaching practices. Finally, the study did not examine how institutional resources, funding models, or participants’ socioeconomic circumstances might affect the implementation of FCL activities.

## Data Availability

The original contributions presented in the study are included in the article/supplementary material, further inquiries can be directed to the corresponding author.
