# Correction: A comparative analysis of traditional classroom and future classroom lab in mathematics education: exploring pre-service teachers' emotions and self-efficacy beliefs

**DOI:** 10.3389/fpsyg.2026.1998246

**Published:** 2026-09-25

**Authors:** Ana Isabel Montero-Izquierdo, Jin Su Jeong, David González-Gómez

**Affiliations:** Department of Science and Mathematics Education, Teacher Training College, University of Extremadura, Cáceres, Spain

**Keywords:** mathematics education, future classroom lab (FCL), pre-service teachers (PSTs), emotions, self-efficacy beliefs, traditional classroom (TC)

An incorrect **Funding** statement was provided. The correct **Funding** statement reads:

“The authors declared that financial support was received for this work and/or its publication. This research was funded through project PID2025-168206OB-100 by the Spanish Ministry of Science, Innovation and Universities and the State Research Agency (MICIU/AEI/10.13039/501100011033), and the ERDF/EU.”

An incorrect **Acknowledgments** statement was provided. The correct **Acknowledgments** statement reads:

“The authors express their gratitude to project PID2025-168206OB-100 by the Spanish Ministry of Science, Innovation and Universities and the State Research Agency (MICIU/AEI/10.13039/501100011033), and the ERDF/EU.”

The original version of this article has been updated.

